# Predicting the Clinical Outcome of Allogeneic Hematopoietic Stem Cell Transplantation: The Long and Winding Road toward Validated Immune Biomarkers

**DOI:** 10.3389/fimmu.2013.00071

**Published:** 2013-03-25

**Authors:** A. Forcina, M. Noviello, M. R. Carbone, Chiara Bonini, Attilio Bondanza

**Affiliations:** ^1^Experimental Hematology Unit, San Raffaele Scientific InstituteMilan, Italy

**Keywords:** allogeneic hematopoietic stem cell transplantation, immune reconstitution, biomarkers, opportunistic infections, graft-versus-host disease

## Abstract

The clinical outcome of allogeneic hematopoietic stem cell transplantation (HSCT) is strongly influenced from the potential complications arising during the delicate phase of post-transplant immune restoration. The quantitative aspects of immune-cell repopulation after HSCT and the qualitative features their functional restitution have been extensively reported. Nevertheless, measurable immune biomarkers predicting the clinical outcome of HSCT await formal validation. The aim of this review is an appraisal of most studies published so far on the predictive value of different T and NK-cell biomarkers after HSCT with emphasis on defined thresholds endorsed by multivariate analysis.

## Introduction

The restoration of a functional immune system is one of the main factors influencing the clinical outcome of allogeneic hematopoietic stem cell transplantation (HSCT). The post-transplant period is characterized by multiple immune defects that expose the patient to a high risk of opportunistic infections and, eventually, disease relapse. The duration of this period may vary according to several variables, including patient age and immune status before transplant, the degree of donor compatibility, the intensity of the conditioning regimen, the source of stem cells, eventual graft manipulation, and pharmacological immune suppression. The normalization of granulocytes, monocytes, and NK-cell numbers usually occurs within the first weeks and, with the possible exception of NK-cells, coincides with their full competence. Conversely, the normalization of T and B cell numbers may take much longer and does not necessarily associate with their immediate functional restitution (Shiobara et al., 1982). Although often used interchangeably, it is therefore important to distinguish between *immune* *reconstitution*, which refers to quantitative immune-cell repopulation, and *immune recovery*, which pertains to their qualitative restitution.

The quantitative reconstitution of T cells post-transplant occurs through two main mechanisms: (i) the early peripheral expansion of donor-derived memory T cells present in the graft, which happens in weeks (ii) the late emergence of host-tolerant naïve T cells originating from donor stem cells after thymic education, which, depending on donor age, occurs in months (van den Brink et al., 2004). The qualitative recovery of T cells may follow their quantitative reconstitution with a delay of many years and implies *de novo* pathogen encounter, with ensuing differentiation into effector and memory T cells.

Although the quantitative aspects of immune reconstitution post-transplant and the qualitative features of immune recovery have been the subject of several studies, a tight association between measurable immune biomarkers and the clinical outcome of HSCT, is currently missing. So far, the specific issue of validating thresholds of immune measurements that may help predicting the incidence of major post-transplant events, such as opportunistic infections, graft-versus-host disease (GVHD), and disease relapse, has been approached by single-center, necessarily small-sized studies. While sometimes sufficiently powered to obtain statistically significant results, these studies often failed to draw definitive conclusions that may be relevant to daily clinical practice. Conversely, the lack of harmonized methods for immune biomarker measurements and the great heterogeneity of the transplant populations between the different studies have prevented from meaningful meta-analysis.

The aim of this review is an appraisal of the studies published so far on the predictive value of different T and NK-cell biomarkers after HSCT with emphasis on the thresholds chosen for statistical analysis. A comprehensive Table 1 detailing the main results from the different studies has also been included. Descriptive studies based on the comparison between different groups as the only statistical approach, studies in the setting of autologous HSCT and immune biomarkers whose predictive value has not been endorsed by multivariate analysis, have been purposely excluded from this review.

**Table 1:** **T- and NK-cell biomarkers, thresholds, and clinical outcome after HSCT**.

Biomarker	Pts	Donor	Days	Statistics	Threshold	Outcome	Reference
ALC	138	HLA-sib	+30	Median	>690 cells/μL	↓TRM ↑LFS ↑OS	Montero et al. (2006)
	157	HLA-sib	+30	Median	>450 cells/μL	↓TRM ↓RI ↑OS	Savani et al. (2007a)
	102	MUD	+30	Arbitrary	>1000 cells/μL	↓TRM ↑LFS ↑OS	Le Blanc et al. (2009)
	65	Haplo UCB	+60	Arbitrary	>1000 cells/μL	↓TRM ↑OS	Ciurea et al. (2011)
	360	UCB	+30	From Savani et al. (2007a)	>200 cells/μL	↓TRM ↑LFS ↑OS	Burke et al. (2011)
CD4^+^ T cells	69	HLA-sib MUD	+90	Arbitrary (HIV)	>200 cells/μL	↓TRM ↑OS ↓infect.	Kim et al. (2006)
	758	HLA-sib MUD	+35	Median	>86 cells/μL	↓TRM	Berger et al. (2008)
	345	HLA-sib MUD	+90	From Matthews et al. (2010)	>200 cells/μL	↓TRM	Buhlmann et al. (2011)
	99	HLA-sib MUD	+20	ROC	>115 cells/μL	↓TRM	Fedele et al. (2012)
CD8^+^ T cells	32	HLA-sib MUD	+365	Arbitrary	>V percentile	↑OS	Koehl et al. (2007)
iNKT/T ratio	71	HLA-sib MUD	+15	ROC	>0.58 × 10^−3^	↓aGVHD ↓TRM ↑OS	Rubio et al. (2012)
	22	Haplo	+545	Median	>10^−4^	No relapse	Casorati et al. (2012)
TREC values	102	HLA-sib	Pre-tx	Categories	172/150,000 T	↑OS ↓infections	Clave et al. (2005)
	33	Haplo	+180	Categories	sj < 0.1/150,000 T; β< 0.001/150,000 T	↑RI	Clave et al. (2012)
Tregs frequencies	60	HLA-sib MUD	aGVHD	Median	>0.5% over TNC	↓TRM ↑OS	Magenau et al. (2010)
	57	HLA-sib MUD	cGVHD	Categories	>3% over PBL	No cGVHD	Koreth et al. (2011)
CMV-specific CD8^+^ T cells	24	HLA-sib MUD	+100	Arbitrary	>10 tet^+^ cells/μL	No CMV disease	Cwynarski et al. (2001)
	83	HLA-sib MUD	+65	Categories	>7 tet^+^ cells/μL	↓CMV disease	Gratama et al. (2010)
	133	HLA-sib MUD UCB	+120	Categories	>1 cyt^+^ cells/μL	↓CMV DNAemia	Tormo et al. (2011)
	131	HLA-sib MUD Haplo	+365	ROC	>3 cyt^+^ cells/μL	No CMV DNAemia	Lilleri et al. (2012)
CMV-specific CD4^+^ T cells	30	HLA-sib MUD	+120	Arbitrary	>2.5 S.I.	↓CMV disease	Krause et al. (1997)
	32	HLA-sib MUD	+40	Median	>0.4 cyt^+^ cells/μL	No CMV DNAemia	Pourgheysari et al. (2009)
	133	HLA-sib MUD UCB	+120	Categories	>1.2 cyt^+^ cells/μL	↓CMV DNAemia	Tormo et al. (2011)
	117	UCB	+30	Arbitrary	>7 S.I.	No infections ↑ LFS	Parkman et al. (2006)
	131	HLA-sib MUD Haplo	+365	ROC	>1 cyt^+^ cells/μL	No CMV DNA	Lilleri et al. (2012)
EBV-specific T cells	33	MUD	+56	Categories	>1 cyt^+^ cells/μL	No relapse	Hoegh-Petersen et al. (2012)
NK-cells	43	Haplo	+15	Median	>9.27 cells/μL	↑LFS	Chang et al. (2008)
	54	HLA-sib	+30	Median	>150 cells/μL	↓TRM ↓RI ↑OS ↓aGVHD	Savani et al. (2007b)
	345	HLA-sib MUD	+365	From Ruggeri et al. (2002)	>150 cells/μL	↓TRM	Buhlmann et al. (2011)

*ALC, absolute lymphocyte count; HLA-sib, HLA-identical sibling; TRM, transplant-related mortality; LFS, leukemia-free survival; OS, overall survival; RI, relapse incidence; MUD, matched unrelated donor; Haplo, HLA-haploidentical; UCB, umbilical cord-blood; ROC, receiver operating characteristic curve analysis; aGVHD, acute graft-versus-host-disease; TREC, T cell receptor excision circles; pre-tx, pre-transplant; TNC, total nucleated cells; cGVHD, chronic graft-versus-host-disease; PBL, peripheral blood lymphocytes; Tet^+^, tetramer-positive cells; cyt^+^, intracellular cytokine-positive; S.I., stimulation index*.

## T Lymphocytes and Invariant NKT Cells

The absolute lymphocyte count (ALC) derived from routine blood-cell testing has been the first immune biomarker explored for predicting the clinical outcome of HSCT. In patients undergoing T cell-depleted HSCT from an HLA-identical sibling (HLA-sib), an ALC above the median [>690/μL (Montero et al., 2006) or >450/μL (Savani et al., 2007a)] at day 30 post-transplant was found to be independently associated with a lower transplant-related mortality (TRM) and longer leukemia-free survival (LFS) and overall survival (OS). The association between an higher ALC at early time points after transplant and a favorable clinical outcome was confirmed by taking an arbitrary threshold of 1000/μL in T cell-replete HSCT from matched unrelated donors (MUD) (Le Blanc et al., 2009) and CD34-selected HSCT form HLA-haploidentical donors (Ciurea et al., 2011), or by taking an arbitrary threshold of 200/μL in umbilical cord-blood (UCB) transplantation (Burke et al., 2011). Investigating the same issue in patients that received a reduced-intensity regimen, however, has found conflicting results (Matthews et al., 2010; Burke et al., 2011), suggesting that the type of conditioning may influence the predictive value of the ALC.

The predictive value of T lymphocyte subsets assessed by flow cytometry, rather than the simpler ALC, has been examined in more sophisticated studies. At day 90 after T cell-replete HSCT form a HLA-sib or a MUD, a CD4^+^ T cell count above 200/μL, a threshold derived from the HIV field, was independently associated with a lower NRM, less opportunistic infections, and a longer OS (Kim et al., 2006). The role for a rapid reconstitution of CD4^+^ T cells in protecting from transplant morbidity and mortality was confirmed in three subsequent studies using slightly different approaches for statistical analysis. The first study found that at day 30 post-transplant, a CD4^+^ T cell count above the median (>86/μL) was associated with a lower TRM (Berger et al., 2008). The second study confirmed the association and observed no impact on relapse incidence (Buhlmann et al., 2011). The third study used receiver operator curve (ROC) analysis of CD4^+^ T cell counts at day 20 post-transplant for determining a threshold of 115/μL, which was retrospectively found to be associated with a lower TRM (Fedele et al., 2012).

Differently from CD4^+^ T cells, the predictive value of CD8^+^ T cell biomarkers is less studied. In a combined series of HLA-sib, MUD, or HLA-haploidentical pediatric HSCT, reaching a CD8^+^ T cell count above the fifth percentile of age-matched controls within the first year post-transplant was found to be independently associated with a longer OS and a trend toward a lower relapse incidence (Koehl et al., 2007).

The pattern of invariant natural killer T cells (iNKT) reconstitution has been explored for predicting the clinical outcome at earlier time points after HSCT and independently from conventional T cells. The reconstitution of iNKT cells after HLA-sib or MUD HSCT was found to precede that of T and NK-cells (Rubio et al., 2012). Accordingly, at day 15 post-transplant an iNKT/T cell ratio above 0.58 × 10^−3^, a threshold identified after retrospective ROC analysis, was associated with a zero likelihood of GVHD. Moreover, reaching an iNKT/T cell ratio above 10^−3^ within the first 3 months after transplantation was independently associated with a lower NRM and a longer OS. In a concomitant study, reaching an NKT/T cell ratio above 10^−4^ within the first 18 months after CD34-selected HLA-haploidentical pediatric HSCT associated with the maintenance of disease remission in all children (de Lalla et al., 2011; Casorati et al., 2012)_._

## TREC Analysis

The molecular analysis of TCR excision circles (TRECs) in circulating T cells allows to quantitatively assess host thymic function, a parameter that has been shown to play a fundamental role in the rapidity of T cell immune reconstitution after HSCT (Talvensaari et al., 2002). After categorization of data from a retrospective cohort, the group of Antoine Toubert has prospectively shown that a pre-transplant TREC content above the threshold of 172 per 150,000 CD3^+^ T cells is an independent factor associated with less infections, including Cytomegalovirus (CMV) reactivation, and a longer OS after T cell-replete HLA-sib HSCT (Clave et al., 2005). The same group has found that at 6 months after CD34-selected HLA-haploidentical pediatric HSCT, a TREC value below detection levels (<0.1 per 150,000 CD3^+^ T cells for sjTREC and <0.001 per 150,000 CD3^+^ T cells for βTREC) was associated with a higher relapse incidence (Clave et al., 2012).

## Natural Tregs

In animal models of HSCT, natural regulatory T cells (Tregs) have a key role in promoting tolerance and, in particular, in protecting from GVHD (Nguyen et al., 2006). In humans, however, there are a number of controversial issues that so far have prevented from confirming the value of Tregs assessment for predicting the risk of GVHD, its grading and response to therapy. These include how to discriminate Tregs from activated T cells and what is the most appropriate way to express Tregs measurements.

In patients with acute GVHD after HLA-sib or MUD HSCT, Tregs frequencies measured at disease onset as the percentage of CD4^+^CD25^bright^Foxp3^+^ T cells over total nucleated cells were reported to inversely correlate with acute GVHD grading (Magenau et al., 2010). Moreover, Tregs frequencies above the median, i.e., >0.5%, were associated with complete response to first-line therapy, resulting in a lower TRM and a longer OS. In another study considering patients with gastrointestinal GVHD, however, peripheral blood as well as mucosal Tregs frequencies, measured as the percentage of CD4^+^ co-expressing Foxp3, were not found to correlate with disease severity (Lord et al., 2011).

The evaluation of Tregs biomarkers for predictive purposes has also yielded conflicting results in chronic GVHD. Some authors have found a paradoxical increase in Tregs measured both as the percentage and as the absolute count of CD4^+^CD25^bright^ (Clark et al., 2004). These T cells were later found to be suppressive *ex vivo*, ruling out that they were activated T cells in disguise. On the contrary, other authors have reported that Tregs frequencies measured as the percentage of CD4^+^CD25^bright^ T cells over peripheral blood lymphocytes below 3%, a threshold derived from linear and logistic regression, were associated with chronic GVHD. This threshold was derived from linear regression models based on data from healthy donors (Zorn et al., 2005). In a phase I/II trial investigating the administration of low dose IL-2 in chronic GVHD, the same group has found that changes in the median count of Tregs somewhat correlated with the probability of responding to the treatment (Koreth et al., 2011).

## Pathogen-Specific T Cells

The value of pathogen-specific T cell responses as an immune biomarker predictive of the risk and the severity of opportunistic infections after HSCT is still controversial. This is mostly due to the use of different methods for measurement (MHC-peptide tetramers, intracellular cytokine staining, ELISPOT assays) and to the lack of harmonized protocols between the different studies. Other contentious issues are whether it is sufficient assessing either CD8^+^ or CD4^+^ responses or it is needed considering both, and whether complex and costly biomarkers are worthy compared with easier, cheaper, and already validated tests, such as serology (Ljungman et al., 2003).

Since CMV disease is a major complication after HSCT, the majority of the studies have focused their attention on CMV-specific responses. In an early study investigating the use of tetramers, it was found that reaching 10 CMV-specific CD8^+^ T cells/μL within the first 100 days after HSCT from an HLA-id or a MUD with discordant serology associated with a zero likelihood of CMV disease (Cwynarski et al., 2001). The predictive value of the CMV-specific CD8^+^ T cell count measured with tetramers was confirmed in a multicenter, prospective study including HLA-sib and MUD HSCT where, after categorization, it was found that a value above the threshold of 7 cells/μL associated with a lower incidence of CMV disease (Gratama et al., 2010).

Other studies have examined the predictive value of CMV-specific CD4^+^ T cell responses showing comparable results. In a pioneering study by the group of Hermann Einsele, it was found that a positive CD4^+^ T cell proliferative response defined as a stimulation index above the arbitrary value of 2.5 within the first 120 days after HLA-sib or MUD HSCT associated with a reduced incidence of CMV disease (Krause et al., 1997). The predictive value of studying CD4^+^ T cell responses was confirmed by using intracellular cytokine staining. In a similar setting, reaching 0.4 CMV-specific CD4^+^ T cells/μL within day 30 and 50, for example, was found to associate with complete prevention from subsequent CMV reactivation (Pourgheysari et al., 2009). In another study, a positive proliferative response to either CMV, HSV, or VZV defined as a stimulation index above 7 was associated not only with a zero probability of opportunistic infections, but also with the maintenance of disease remission, indicating that the recovery of pathogen-specific immunity may serve as a surrogate biomarker of immune restoration (Parkman et al., 2006).

In certain studies, the concomitant exploration of both CD4^+^ and CD8^+^ CMV-specific T cells allowed determining the predictive thresholds for the two subsets in a compared manner. In a pediatric population, after ROC analysis of intracellular cytokine staining data from a retrospective cohort (Lilleri et al., 2006), it was found that reaching a CMV-specific T cell count above the threshold of 1/μL for CD4^+^ T cells and of 3/μL for CD8^+^ T cells within the first year after HLA-sib, MUD, or HLA-haploidentical pediatric HSCT associated with a zero likelihood of CMV reactivation up to 2 years thereafter (Lilleri et al., 2012). These thresholds were found to be remarkably similar (1.2 and 1/μL for CD4^+^ and CD8^+^ CMV-specific T cells, respectively) in HLA-sib and MUD adult HSCT (Tormo et al., 2011) suggesting that measuring pathogen-specific responses in both subsets may be of predictive value, although with slightly different thresholds.

The results of the studies on Epstein-Barr virus (EBV)-specific responses are more controversial. Although measuring EBV-specific T cell responses by intracellular cytokine staining was found to have no value in predicting the likelihood of post-transplant lymphoproliferative disease (Hoegh-Petersen et al., 2011), finding at day 56 an EBV-specific T cell score above 1, a threshold found after categorization of a complex measure including CD4 and CD8 viral epitopes, was associated with a near-zero likelihood of leukemia relapse (Hoegh-Petersen et al., 2012).

## NK-Cells

The discovery that NK alloreactivity plays a major role in preventing disease relapse after CD34-selected HLA-haploidentical HSCT (Ruggeri et al., 2002), has fostered a number of studies investigating the predictive value of NK-cell biomarkers on clinical outcome. The reconstitution of NK-cells post-transplant is slightly slower compared to other cells of the innate immune system, but definitively faster than conventional T cells. At 30 days after T cell-depleted HLA-sib HSCT, an NK-cell count above the median (>150/μL) was found to associate with less acute GVHD, a lower relapse incidence, and a longer OS (Savani et al., 2007b). The association between higher NK-cell counts and lower relapse incidence was however restricted to patients with myeloid leukemia, a disease that is susceptible to NK lysis. The predictive value of NK-cell counts at early time points post-transplant was confirmed in the setting of unmanipulated HLA-haploidentical HSCT, where, after categorization, an NK-cell count above the threshold of 9.27/μL as early as 15 days post-transplant was associated with a longer LFS (Chang et al., 2008). The picture was shown to differ in the context of T cell-replete HSCT, where higher NK-cell counts (>150/μL, a threshold taken from previous studies (Savani et al., 2007b) were associated with a lower TRM at late time points, but not with a lower relapse incidence (Buhlmann et al., 2011).

## Conclusion

In the era of predictive and molecular medicine, the practice of HSCT is still characterized by many prognostic uncertainties. Since many complications of HSCT derive from the state of temporary, although often prolonged, state of immunodeficiency post-transplant, it is clear that finding a tight correlation between certain immune system defects and the different complications may help predict the overall clinical outcome. This is important not only for improving the care of patients, who may expect benefits from ready and tailored strategies of intervention, such as intensification or discontinuation of antimicrobial and immune suppressive drugs, but also for establishing accepted surrogate markers of immune restoration that may accelerate the clinical development of novel transplant strategies, including the transfer of pathogen-specific T cells generated after *ex vivo* stimulation (Feuchtinger et al., 2010; Heslop et al., 2010).

The road leading to the validation of immune biomarkers answering to this crucial, unmet need is long and winding, and is possibly better traveled by joining forces in multicenter efforts. The recent launch of different, retrospective, and prospective studies coordinated by the Immunobiology Working Party of the European Bone Marrow Transplantation society goes exactly in this direction and is expected to contribute to filling this gap in the near future.

## Conflict of Interest Statement

The authors declare that the research was conducted in the absence of any commercial or financial relationships that could be construed as a potential conflict of interest.
